# Optimal User Association Strategy for Large-Scale IoT Sensor Networks with Mobility on Cloud RANs

**DOI:** 10.3390/s19204415

**Published:** 2019-10-12

**Authors:** Taewoon Kim, Chanjun Chun, Wooyeol Choi

**Affiliations:** 1 School of Software, Hallym University, Chuncheon 24252, Korea; taewoon@hallym.ac.kr; 2 Future Infrastructure Research Center, Korea Institute of Civil Engineering and Building Technology, Goyang 10223, Korea; chanjunchun@kict.re.kr; 3 Department of Computer Engineering, Chosun University, Gwangju 61452, Korea

**Keywords:** Cloud RAN, user association, handover, load balance, IoT Sensor, mobility, wireless networks, optimization

## Abstract

In networking systems such as cloud radio access networks (C-RAN) where users receive the connection and data service from short-range, light-weight base stations (BSs), users’ mobility has a significant impact on their association with BSs. Although communicating with the closest BS may yield the most desirable channel conditions, such strategy can lead to certain BSs being over-populated while leaving remaining BSs under-utilized. In addition, mobile users may encounter frequent handovers, which imposes a non-negligible burden on BSs and users. To reduce the handover overhead while balancing the traffic loads between BSs, we propose an optimal user association strategy for a large-scale mobile Internet of Things (IoT) network operating on C-RAN. We begin with formulating an optimal user association scheme focusing only on the task of load balancing. Thereafter, we revise the formulation such that the number of handovers is minimized while keeping BSs well-balanced in terms of the traffic load. To evaluate the performance of the proposed scheme, we implement a discrete-time network simulator. The evaluation results show that the proposed optimal user association strategy can significantly reduce the number of handovers, while outperforming conventional association schemes in terms of load balancing.

## 1. Introduction

Utilizing short-range, low-power base stations (LPBSs) for wireless communication has been considered as a breakthrough in the information and communication technology (ICT) field. Although the conventional cell towers or macro base stations (macro BSs or MBSs) have advantages such as large coverage, they are associated with high operating expenses (OPEX) and capital expenditure (CAPEX) for their maintenance and installation, respectively, as well as low cell-edge user throughput. Therefore, LPBSs have been considered as an alternative solution to solve these issues. Their key advantage is the short distance between the transmitter and receiver that guarantees the high channel gain. Moreover, the short coverage increases the spatial reuse [1]. This technology was widely discussed in relation to third generation partnership program (3GPP) long-term evolution (LTE) cellular networking systems in the form of pico-cell and femto-cell, or, more generally, small-cells [2,3]. Consequently, in the era of next generation 5G networking systems, LPBSs have become one of the essential components.

**Figure 1:**
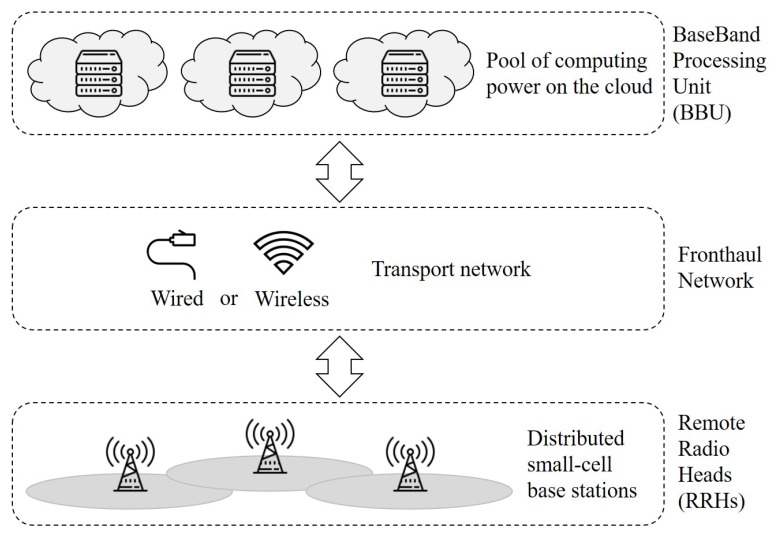
The three-layer architecture of C-RAN.

One of the most evident transformations from the current 4G to the next generation 5G cellular network is the network architecture called cloud-based radio access network (C-RAN). C-RAN [4,5,6,7], in general, has a three-layer structure [8]: the remote radio head (RRH), the fronthaul network, and the baseband processing unit (BBU) as shown in Figure 1. As can be seen in the figure, the general C-RAN architecture utilizes short-rage RRHs to provide the connection and data service to users. As previously mentioned, the short distance between communicating parties yields a reduced power consumption. Owing to collective powerful computing resources provided by the BBU pool, C-RAN is capable of handling a large number of users processing much larger volume of traffic than the 4G configuration with MBSs. To be specific, compared to the current 4G technology, the 5G technology is expected to achieve 1000 times larger mobile data throughput, to handle 10–100 times more user devices, to yield 10 times less power consumption, to enable 1 ms latency, and to have other desirable characteristics [9,10]. The interested readers can refer to [4] for an in-depth technical introduction to the 5G wireless systems.

Such enhancements will lead to further development of emerging applications including a large-scale network of Internet of Things (IoT) [9]. IoT applications are expected to be widely used in many applications related to the concept of smart home/city/factory, opening up wide business opportunities [11]. For example, a corresponding increase in the annual economic impact of USD $2.7–$6.2 trillion is projected by 2025 [11]. However, the gigantic market opportunity is associated with the large amount of burden to the underlying networks in terms of the heavy traffic load and a large number of IoT devices to be connected to the network.

It is expected that in the very near future 212 billion IoT devices are to be connected, and the aggregated traffic from these devices is to account for nearly half of the entire traffic over the Internet [11]. Although the 5G wireless system is a promising platform for the future IoT technologies [4], combined with the C-RAN structure, the projected IoT device influx may result in many challenges. In this study, we particularly consider the following two: the unbalanced traffic load among RRHs and the overflow of control messages.

These two challenges arise from the fact that RRHs are short-ranged, and the mobility of end user devices is inevitable [4]. To provide the coverage, RRHs have to be densely deployed [7], letting many of them be overlapped. Therefore, many users could have access to multiple RRHs to associate with. Considering the fact that IoT traffic is not the only load required to be handled by RRHs, it is possible that if a small number of RRHs happen to be over-populated by IoT devices they will easily become congested. This can be a critical hurdle to maintain real-time, mission critical applications. The situation can become worse in the cases when the IoT devices have mobility. Due to the small coverage of RRHs, even a small change of IoT device’s location can cause a handover (Handover is a procedure of terminating the current association with RRH *i* and then switching to another RRH *j* where i≠j. Data communication between a user device and an RRH becomes possible only after making an association.). Since we consider the case of large-scale IoT devices being deployed and operating, the entire network may be significantly influenced in a negative way from frequent handovers and the resulting control message flood.

In this paper, we propose an optimal user association strategy between mobile IoT devices and RRHs to effectively balance the traffic load between RRHs on a dense C-RAN. Employing such strategy, the number of congested RRHs can be minimized, therefore reducing the network delay. In addition, we propose a novel way of reducing the occurrences of handovers during the communication process. The reduced number of handovers not only allows diminishing the amount of control messages, but also eliminating the amount of time needed to perform unnecessary handover procedures. The specific contributions we make in this work are summarized as follows:

- We propose an optimization problem formulation for the user association, which will allow distributing the traffic load evenly among short-range RRHs to prevent them from being congested.
- We suggest a revised optimal user association strategy, which will allow minimizing the occurrences of handovers to prevent control message flooding. The optimal solution to the revised problem formulation simultaneously enhances the load-balancing performance and minimizes the number of handovers. Such a joint consideration has not been made in previous works yet, which is one of the major contributions we make in this paper.
- We propose a way to reduce the computational load for operating the network with the usage of the proposed optimal user association scheme. In a nutshell, if there is no change in the network topology, the previous association can be re-used without necessity to find an optimal solution.
- We implement a discrete-time network simulator and evaluate the performance of the proposed methods. In addition, we compare the performance of the proposed strategies against the conventional association strategies.
- We monitor and analyze the time complexity of each algorithm to evaluate whether they are applicable for large-scale, real-time applications.

The remainder of this paper is organized as follows. Section 2 summarizes several important related research works. In Section 3, we first introduce the network parameters, and then, we propose the optimal user association problem formulations. Section 4 presents the network parameters and the evaluation results. A brief discussion on the time complexity of the proposed schemes is given in Section 5. Finally, Section 6 concludes this paper. The abbreviations used in this paper are outlined in Table 1.

## 2. Related Work

Optimal user association for networks with short-range BSs have been widely studied in the literature. Kim et al. [3] proposed an optimal user-BS association scheme for LTE with small-cell BSs. They proposed a tractable and distributed solution which minimizes the power consumption from BSs while satisfying users service demand (i.e., traffic requirement). However, this study considers neither the task of load balancing among small-cell BSs nor possible frequent handover problems. In the paper [8], the authors proposed an optimal resource scheduling method for heterogeneous C-RANs. Considering the uncertainties in users’ mobility and their traffic demand, the authors proposed a multi-stage stochastic programming based optimal solution that minimizes the overall power consumption from BSs while maximizing the profit to network service operators. However, the proposed solution does not consider the need for load balancing or preventing control message flooding due to frequent handovers.

Load-aware or load-balancing association schemes have also been studied in previous research works. Zhang et al. [12] proposed an optimal user association and power adjustment scheme for ultra-dense wireless networks. Along with maximizing the network throughput-energy efficiency, the proposed scheme considers the traffic load handled by BSs by means of the interference level. However, the proposed scheme does not consider the possible performance deterioration caused by frequent handovers. The authors of [13] proposed an optimal user association scheme for load balancing in heterogeneous cellular networks. After forming a network-wide weighted utility maximization problem, the authors transformed it into a distributed algorithm of a low complexity. However, there is no discussion on the issue of frequent handovers, which should not be overlooked in dense networks such as C-RAN.

The importance of load-balancing mechanisms in wireless networks has been discussed in detail in [14]. As it is an effective way of enhancing the resource utilization, fairness, delay performance, throughput, and other important aspects of mobile networks, there have been many efforts made to investigate this topic, including the study discussed in [15]. It has an initial approach similar to our proposed scheme; however, their solution for wireless multi-hop networks cannot be applied to C-RAN which is of our interest in this work. Ye et al. [16] proposed a load-aware user-cell association method for load balancing in small cell assisted heterogeneous cellular networks. By using the decomposition technique, the authors proposed a distributed algorithm of low complexity to be applicable in real-time applications. However, the proposed scheme does not consider the effect of frequent handovers, which can cause severe network congestion in dense networks such as C-RAN. Jin et al. [17] proposed a low-complexity algorithm to maximize the network energy efficiency with a fairness constraint for a dense heterogeneous network. To achieve this goal, the authors designed a joint small cell on/off control and load-balancing method aimed to optimize each task separately. Despite of the proposed scheme’s practicality in heterogeneous networks, it is not applicable to the C-RAN network considered in this research. This is due to the fact that coverage holes may occur in CRANs if some RRHs become unavailable [18].

In addition, there have been studies trying to reduce the number of handovers using an advanced transmission coordination scheme such as coordinated multi-point transmission, or transforming a generic C-RAN into a heterogeneous C-RAN [19]. However, such approach can significantly increase the computational complexity of the user association procedure. A signaling overhead problem caused by handovers is discussed in detail in [20] focusing on heterogeneous networks with small cell BS. The authors proposed a simple handover scheme, which differentiates users with respect to their speed, consequently determining whether or not to allow users to connect to a small cell BS or not. However, this strategy does not apply to the conventional C-RAN model. In addition, under some circumstances, where many low-speed users are located close to each other, the nearby small cell BS may experience network congestion or buffer overflow.

In [21], the authors proposed a resource optimization method for multi-cell OFDMA systems under the backhaul constraints. By allowing BSs to cooperate and share channel state information (CSI) with each other, their proposed solution allocates network resources to maximize the energy efficiency. The initial non-convex problem is transformed by exploiting the fractional programming, and then, the proposed iterative algorithm is applied to find a sub-optimal solution. However, their proposed solution makes an assumption that CSI is perfectly known and shared among BSs. Such an assumption is hard to guarantee in practice, incurs delay for collecting CSI, and increases computational overhead in resource optimization. Thus, it is not applicable to some real-time applications. In addition, the network setting assumed in [21] cannot be directly applied to C-RAN.

In addition, the feasibility of the C-RAN with software-defined network was shown by Yang et al. [22,23,24] via empirical experiments. The authors proposed a cloud-based radio over optical fiber network (C-RoFN) architecture with multi-stratum resource optimization (MSRO) [22,23]. With the OpenFlow, which is the enabler of the software-defined network (SDN), they implemented a testbed to show that the proposed C-RoFN can orchestrate multi-layer resources (i.e., radio frequency, optical spectrum and BBU processing resources) to enhance radio coverage as well as to fulfill the QoS requirements. In [24], the authors studied a multi-dimensional resource integration (MDRI) technique for service provisioning in C-RoFN. Through an empirical study, the authors showed that MDRI can enhance both the end-to-end responsiveness and the radio coverage.

In contrast to the previous studies on load balancing and/or handover-aware association strategies, the methods proposed in this work have the following advantages and distinctions. We consider dense C-RAN networks with large-scale mobile IoT devices. As pointed out in [25], it is one of the most important scenarios in the 5G system. In contrast to other studies conducted on user association, which focused only on either load balancing or handovers (or even neither of these two), we jointly consider both tasks. Such approach is important when dense C-RANs with mobile users are considered. In addition to proposing a simple, yet effective technique to reduce the time complexity, we conducted an evaluation to show that the proposed algorithms can be used in real-time under certain types of networks. We also investigated a possible solution to significantly reduce the time complexity. We evaluated and analyzed the performance of the proposed schemes as well other conventional ones under different scenarios (i.e., with different speed or the number of mobile IoT devices) to understand how the proposed methods behave under diverse network settings.

## 3. Problem Formulation

In this paper, we consider a C-RAN with densely deployed RRHs. We assume a fronthaul network with wired and possibly optical connections [22], and the IoT devices in the network are mobile. We assume a typical delay-tolerant IoT monitoring application, in which IoT devices transmit fixed-length, small-sized data (or packets) to their associated RRHs at a predefined lowest and constant rate, referred to as robust rate. The rate is robust, meaning that as long as an IoT device is located within the transmission range of its associated RRH, the rate is guaranteed regardless of the interference from nearby IoT transmitters if any. The specifics as to how the robust rate is guaranteed and what happens if not will be addressed shortly. We focus on the uplink transmission in this work; in this case, IoT devices upload their sensed readings or recorded measurement data to their associated RRHs. Time is assumed to be divided into fixed-length slots, and an IoT device can associate with a single RRH at each time slot. On the other hand, an RRH can associate with multiple IoT devices at the same time.

To upload data, an IoT device needs to associate with an RRH. Once the association procedure is completed, the IoT device can transmit data to its serving/associated RRH each time slot. We assume that the proximity of an IoT device to nearby RRHs solely determines the possibility of communication and association. That is, if an IoT device *i* is within the transmission range of an RRH *r*, *i* can associate and communicate with *r*. Since RRHs are densely deployed, it is likely that some IoT devices are located within the coverage of multiple RRHs at the same time. We assume that IoT device *i* is capable of transmitting $l_{i}$ number of data packets within a single time slot if the associated RRH has enough space in its buffer.

For each time slot, information on the proximity between IoT devices and RRHs is collected and uploaded to BBU. It can be easily and quickly done via simple cooperation between RHHs and IoT devices. Each RRH *r* periodically broadcasts short pilot signals at a predefined transmit power, and those IoT devices, which are close enough to the RRH, will be informed that they are within the transmission range of RRH *r*. Upon receiving the pilot signal from a nearby RRH, each IoT device calculates the downlink channel gain with the known transmit power. Although the wireless link is mostly asymmetric, each IoT device takes the gain as a prediction for the uplink, and uses the gain to check if it can transmit data at the robust rate in the uplink. If it can, the IoT device puts the RRH in the nearby-RRH list. However, the uplink transmission still can fail since the uplink channel gain is a prediction and the IoT devices are mobile. In such cases, the automatic repeat request function or its variants [26] can help to guarantee the successful data delivery. Once the pilot broadcasting period is over, IoT devices upload the list of nearby RRHs to BBU. After collecting such lists, BBU defines a proximity matrix, $N={\left[n_{r,i}\right]}_{\left\{r\in R,i\in I\right\}}$, where $n_{r,i}$ equal to 1 or 0 indicates RRH *r* has device *i* within its coverage or not, respectively; R is the index set of RRHs; and I is the index set of IoT devices. Hereinafter, N is assumed to be given to (or defined for) each optimization problem.

RRHs are homogeneous, and, therefore, they have the same traffic processing capacity, *c*, per a time slot (RRHs may also have another capacity constraint, limiting the number of users to associate with at the same time. In this work, however, we assume that such constraint is indirectly included in *c*. For example, *c* can take the minimum value between the RRH’s data processing capacity and the fronthaul link capacity.). That is, each RRH can process *c* number of data packets within each time slot. Since IoT devices are not the only users uploading their traffic, each RRH *r* has $b_{i}$ amount of background traffic (i.e., the number of data packets buffered and to be processed in the given time slot). Before we introduce our proposed algorithms in detail, we provide the brief summary as follows:

- ALGO-1 evenly distributes the traffic load among RRHs by solving P. (2).
- ALGO-2 evenly distributes the traffic load and minimizes the number of occurring handovers by solving P. (3).
- ALGO-3 is basically equivalent to ALGO-2 except for the fact that it reuses the previous optimal association decision if there is no change in the network topology (i.e., the proximity between IoT devices and RRHs).

Notations used in this paper are summarized in Table 2. Bold and uppercase letters indicate matrices, vectors are denoted by bold and lowercase letters, and calligraphic fonts are used to indicate sets.

### 3.1. Optimal Load-Balancing Association

We begin with introducing an optimal load-balancing association strategy. As mentioned above, evenly distributing the traffic load among RRHs is essential to prevent congestion and to reduce network delay (Reducing the network delay is essential for C-RANs. Since passing the signals upward/downward via the fronthaul network already requires a certain amount of time, to guarantee the target network delay defined in 5G, it is important to prevent network congestion as much as possible, therefore aiming to avoid causing any additional delay.). The optimal load-balancing problem is formulated as below (called P. (1)):

(1a) $$\begin{array}{cc} \min_{X}.\;\; & \max\{b_{r}+\sum_{\forall i\in I}l_{i}\times x_{r,i},\forall r\in R\}\\ \mathrm{subject}\;\text{to}:\;\; & \end{array}$$

(1b) $$\begin{array}{cc} \forall r\in R:\;\; & b_{r}+\sum_{\forall i\in I}l_{i}\times x_{r,i}\leq c \end{array}$$

(1c) $$\begin{array}{cc} \forall i\in I:\;\; & \sum_{\forall r\in R}x_{r,i}=1 \end{array}$$

(1d) $$\begin{array}{cc} \forall r\in R,\forall i\in I:\;\; & 0\leq x_{r,i}\leq n_{r,i} \end{array}$$

(1e) $$\begin{array}{cc} \forall r\in R,\forall i\in I:\;\; & x_{r,i}\;\text{binary} \end{array}$$

The problem formulation is quite straightforward. The one and only decision variable is $X={\left[x_{r,i}\right]}_{r\in R,i\in I}$. As defined in Equation (1e), $x_{r,i}$ is binary indicating whether RRH *r* is associated with IoT device *i* (i.e., $x_{r,i}=1$) or not (i.e., $x_{r,i}=0$). The objective function in Equation (1a) minimizes the largest traffic load handled by individual RRHs. Consequently, portions of the traffic handled by different RRHs can become close to each other, hence, balancing the traffic load among them. Equation (1b) states that, for each RRH *r*, the sum of its background traffic and the total traffic from its associated IoT devices cannot exceed the RRH capacity limit. Each IoT device *i* needs to associate with one RRH within a single time slot to satisfy the QoS (Quality of Service) requirement as defined in Equation (1c). IoT device *i* can associate with RRH *r* only when it is close enough to *r*; in other words, the association can happen only if *i* is within the transmission range of *r*, as stated in Equation (2d).

To enable P. (1) to be easily computed by a computer solver, we slightly modify the problem by introducing a new decision variable *k*. Rewriting the resulting problem yields the following problem formulation (called P. (2)), which is equivalent to P. (1):

(2a) $$\begin{array}{cc} \min_{X,k}.\;\; & k\\ \text{subject}\;\text{to}:\;\; & \end{array}$$

(2b) $$\begin{array}{cc} \forall r\in R:\;\; & b_{r}+\sum_{\forall i\in I}l_{i}\times x_{r,i}\leq c \end{array}$$

(2c) $$\begin{array}{cc} \forall i\in I:\;\; & \sum_{\forall r\in R}x_{r,i}=1 \end{array}$$

(2d) $$\begin{array}{cc} \forall r\in R,\forall i\in I:\;\; & 0\leq x_{r,i}\leq n_{r,i} \end{array}$$

(2e) $$\begin{array}{cc} \forall r\in R,\forall i\in I:\;\; & x_{r,i}\;\mathrm{binary}. \end{array}$$

(2f) $$\begin{array}{cc} \forall r\in R:\;\; & b_{r}+\sum_{\forall i\in I}l_{i}\times x_{r,i}\leq k \end{array}$$

(2g) $$\begin{array}{cc} \; & k\;\mathrm{free} \end{array}$$

The first proposed algorithm ALGO-1 makes use of the optimal association decision from P. (2) as follows. For each time slot, the BBU runs the optimization problem to compute the optimal association. The decision is then distributed to RRHs, in such a way that that they can associate with the appointed IoT devices. Finally, IoT devices can transmit the data packets to their associated RRHs.

### 3.2. Optimal Load-Balancing Association with Minimal Handovers

The optimal solution from P. (2) allows achieving the optimal load balancing. In other words, it prevents the case when too much traffic is concentrated in certain RRHs. As mentioned above, it is important to prevent network congestion at such RRHs to avoid increase in the network delay. However, P. (2) does not consider the number of handovers. If the optimal solution is the same, which is lowering *k* in P. (2) as much as possible, the computer solver will choose an arbitrary solution from the ones yielding the same results; the specific behavior may differ depending on the computer solvers.

**Figure 2:**
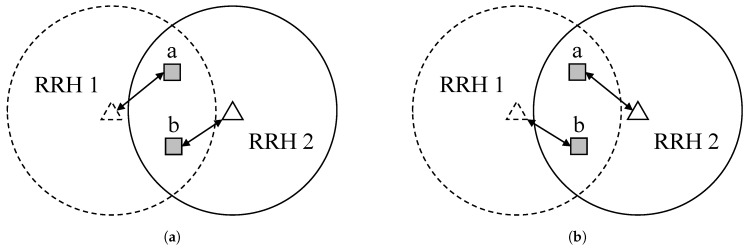
Different but equally optimal load-balancing decisions from the perspective of P. (2). (**a**) Association at time *t*; (**b**) Association at time t+1.

Figure 2 illustrates two different decisions yielding the same optimal solution. RRH 1 and RRH 2 are installed within the network, and their transmission ranges are depicted with a dashed line and a solid line, respectively. In the overlapped region, there are two IoT devices *a* and *b*, which are stationary. Both *a* and *b* are assumed to be transmitting the same number of data packets within each time slot, and both RRHs have the same amount of background traffic. In terms of the optimal objective value, both Figure 2a,b are equally optimal. Therefore, a computer solver may choose Figure 2a as the optimal solution at time *t* and Figure 2b at time t+1. Such a strategy does not deteriorate the quality of the solution, but it incurs two handovers, one for *a* and the other for *b*. In a large-scale network with many mobile IoT devices, this may cause the entire network overloading with numerous unnecessary handovers and the resulting control messages.

To minimize the costs caused by frequent handovers, we propose introducing a revision of P. (2). For each time slot, once the optimal solution is found, we store the optimal solution X. As each element in the matrix is binary, it only requires |R|×|I| bits to store the optimal solution. Thereafter, at time *t*, we take the differences between the previous optimal decision $X_{t-1}$ and the current one $X_{t-1}$ into consideration. Obviously, minimizing the difference between these two decisions will also allow minimizing unnecessary handovers. The revised problem formulation is provided below (called P. (3)):

(3a) $$\begin{array}{cc} \min_{X,k}.\;\; & \alpha \times \frac{1}{c}k+\left(1-\alpha \right)\frac{1}{2|I|}{∥X_{t}-X_{t-1}∥}_{1} \end{array}$$

(3b) $$\begin{array}{cc} \forall r\in R:\;\; & b_{r}+\sum_{\forall i\in I}l_{i}\times x_{r,i}\leq c \end{array}$$

(3c) $$\begin{array}{cc} \forall i\in I:\;\; & \sum_{\forall r\in R}x_{r,i}=1 \end{array}$$

(3d) $$\begin{array}{cc} \forall r\in R,\forall i\in I:\;\; & 0\leq x_{r,i}\leq n_{r,i} \end{array}$$

(3e) $$\begin{array}{cc} \forall r\in R,\forall i\in I:\;\; & x_{r,i}\;\text{binary}. \end{array}$$

(3f) $$\begin{array}{cc} \forall r\in R:\;\; & b_{r}+\sum_{\forall i\in I}l_{i}\times x_{r,i}\leq k \end{array}$$

(3g) $$\begin{array}{cc} \; & k\;\text{free} \end{array}$$

In Equation (3a), α∈[0,1] is a design parameter, and it determines the weight on the two terms. By adjusting the parameter, we can provide a different priority to each term in the objective. In the objective function, the inverses of two constants, i.e., *c* and 2|I|, are multiplied by the first and second terms, respectively, to normalize each term. The first term still has the same variable *k* as defined in P. (2), while the newly added second term measures the differences in association between the current and the previous decisions, $X_{t-1}$ and $X_{t}$, respectively. Being a design parameter, α can take any value within [0,1]. If α is small, the solution focuses more on minimizing the number of handovers than on enhancing the fairness performance. For large values of α, P. (3) will yield the opposite results. To strike a balance between the two options, one can set α to the median value of the range.

The second proposed algorithm ALGO-2 runs similarly to ALGO-1. It computes the optimal association decision from P. (3), which is then used to make associations between RRHs and IoT devices. In Section 4, we show the effects of different values of α on the performance.

The time complexity of P. (2) is no greater than that of P. (3), and both are mixed integer linear programming (MILP) problems, or mixed binary linear programming to be specific. Please note that the second term in the objective function of P. (3) can be transformed to linear inequality equations. Due to the binary variables, the search space of both problems has the size of two to the power of the number of binary variables. As the network size increases, i.e., as the number of RRHs and the IoT devices on the network increases, the problem becomes intractable in general. However, there are some efficient exact algorithms that can quickly solve ILP, such as the variants of branch and bound [27]. In Section 4.4, we show that under the given network settings we can find the optimal solution shortly.

### 3.3. Enhancement for Time Complexity

The BBU pool in the C-RAN architecture carries out all computations, including that of the optimization problem. Due to the high computing power of C-RAN as well as the use of sophisticated methods for handing binary/integer optimization variables, such as branch and bound with sequential linearization, solving the optimization problem does not take a significant amount of time; the results of the time complexity evaluation are discussed in Section 4. However, to achieve the scalability of the proposed methods, we propose a simple addition to the ALGO-2 to reduce the processing time required for the optimal association at the expense of memory space.

As mentioned above, the proximity matrix N is computed to identify which IoT devices are within the transmission range of which RRHs. If we have the matrices for the two consecutive time slots, i.e., $N_{t-1}$ and $N_{t}$, we can identify the changes in the proximity information. The easiest way is to compute the following: ${∥N_{t}-N_{t-1}∥}_{1}=\sum_{\forall r\in R}\sum_{\forall i\in I}\left|n_{r,i}^{t}-n_{r,i}^{t-1}\right|$ where $N_{t}={\left[n_{r,i}^{t}\right]}_{r\in R,i\in I}$ and $N_{t-1}={\left[n_{r,i}^{t-1}\right]}_{r\in R,i\in I}$. At time *t*, if there is no change in $N_{t}$ compared to $N_{t-1}$, the network can reuse the solution $X_{t-1}$ found in the previous time slot. When the IoT devices are stationary or when they are moving at a slow speed, this approach can significantly reduce the computational time. Even in the case when the IoT devices are fast-moving objects, some optimal solutions can still be valid for the next time slot if the length of a time slot is short.

The third proposed algorithm ALGO-3 runs as follows. It stores both the previous optimal association, $X_{t-1}$, and the previous proximity matrix, $N_{t-1}$. For each time *t*, it checks if there are any changes in the proximity information. If ${∥N_{t}-N_{t-1}∥}_{1}=0$, ALGO-3 terminates and reuses $X_{t-1}$, which is the previous optimal association decision. If not, ALGO-3 computes P. (3) to find the optimal association rule with the fewest handovers. ALGO-3 uses a matrix of all 0 values for $N_{0}$.

## 4. Evaluation

### 4.1. Network Configuration

**Figure 3:**
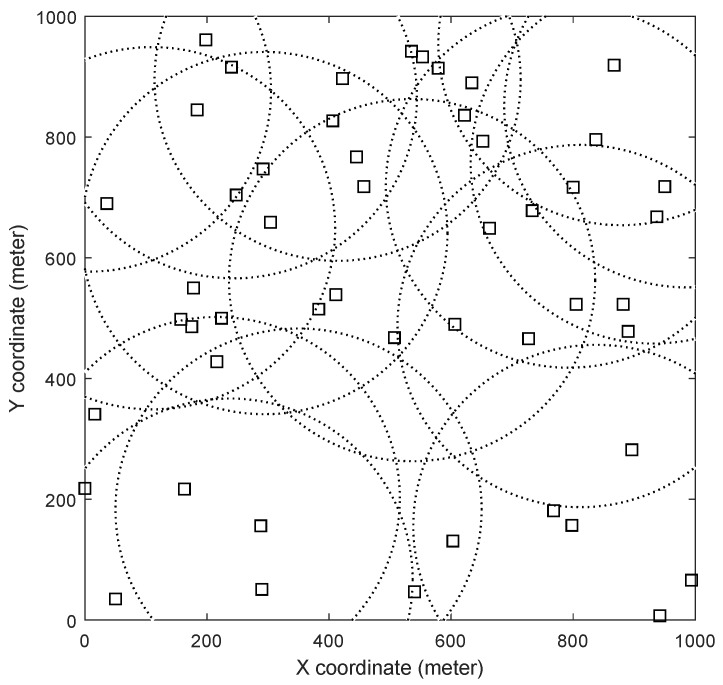
The initial layout of the network with 15 RRHs and 50 IoT devices. Dotted lines indicate the transmission ranges of RRHs and the empty rectangles indicate the initial positions of the IoT devices.

For the purpose of evaluation and comparison, we configured the network as follows. The area of our interest is 1000 m by 1000 m. There are 15 RRHs and 50 devices installed by default, and all of them are randomly located using the uniform distribution. The number of IoT devices established for evaluation is from 50 to 250. The transmission range of each homogeneous RRH is 300 m. Devices on the network keep moving using the random way-point model. The maximum speed of each IoT device, *v*, is chosen between 1 and 100 m/s for each simulation. Then, for each time slot, an IoT device moves at the speed of Uniform(0,*v*) m/s. We specify the specific value of *v* before introducing the evaluation results. Each time slot is 1 s, and each simulation runs for 1000 s. We set *c* to |I| meaning each RRH can handle or process |I| number of data packets per a time slot. Within the current configuration, it is considered to be large enough not to cause any buffer overflow to any of RRHs. The background traffic $b_{r}$ is randomly chosen using the uniform distribution from the set $\left\{1,2,3,\cdots ,\frac{c}{2}\right\}$. The data rate at each IoT device, $l_{i}$, is randomly chosen from the set {1,2,3}. Both $b_{r}$ for all *r* and $l_{i}$ for all *i* remain constant throughout each simulation. The network parameters are summarized in Table 3. The initial layout of the network with 50 IoT devices is depicted in Figure 3.

We used MATLAB [28] to implement the discrete time simulator. To formulate and solve each optimization problem, we used CVX [29] to model the proposed optimization problem formulations and Gurobi [30] as an optimization problem solver. A desktop PC with Intel^®^ Core^TM^ i7-7700 CPU @ 3.60 GHz and 16 GB memory was used for evaluation without graphics processing units (GPU) support. To perform the comparison, we implemented the following two conventional association schemes:

- Shorted-Distance-First association scheme (SDF): Each IoT device associates with the closest RRH. It is an approximation of the highest-channel-gain-first association scheme.
- Random Association scheme (RND): Each IoT device randomly chooses an RRH to associate with.

It should be noted that neither SDF nor RND allows any association between RRH *r* and IoT device *i* if *i* is outside the coverage of *r*. The time complexity of SDF is O(|R|·|I|), while that of RND is either O(|R|·|I|) or O(|R|) depending on implementation.

### 4.2. Load-Balancing Performance

**Figure 4:**
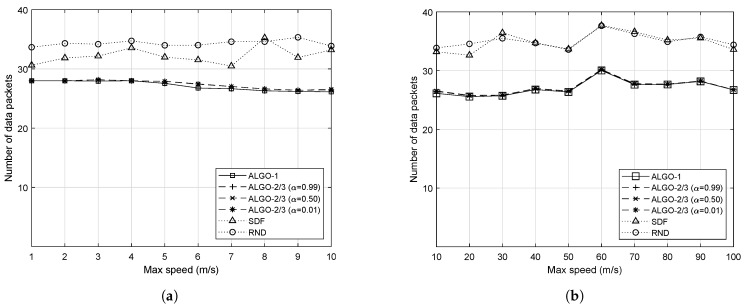
Average maximum load among RRHs with respect to different upper bounds of the speed in a system with 50 mobile IoT devices. (**a**) When IoT devices are moving at a slow speed; (**b**) When IoT devices are moving at a fast speed.

Before we measured the load-balancing performance itself, we first measured the maximum load among RRHs on average, which is defined as follows. For |R| number of RRHs, the maximum load among the RRHs is: $\max\{ld\left(r_{i}\right)\;\mathrm{for}\;\forall i\in R\}$, where $ld(r_{i})$ is a function returning the current traffic load of RRH $r_{i}$. As a reminder, the proposed schemes basically allow minimizing the maximum load among RRHs to achieve optimal load balancing. As the objective value gets smaller, the task of distributing the traffic load from IoT devices evenly among RRHs becomes easier. The evaluation results with respect to the maximum speed of IoT devices are shown in Figure 4. The *x*-axis represents the maximum speed of IoT devices and the *y*-axis depicts the maximum number of packets to be processed by individual RRHs on average.

Regardless of *v*, the proposed schemes, i.e., ALGO-1 and ALGO-2/3, allow achieving the lowest maximum traffic load for individual RRHs. It should be noted that ALGO-2 and ALGO-3 should have similar performance except for the time complexity; thus, we grouped their results together. It is worth mentioning that even the small value of α, i.e., 0.01, does not significantly change the result on the maximum number of data packets to be processed by individual RRHs. Although ALGO-1 shows the best performance overall, the ALGO-2/3 with different α can produce comparable results. The difference between ALGO-1 and ALGO-2/3 became clear when we analyzed the number of handovers that occurred. ALGO-2 and ALGO-3 should have the same performance as they are basically the same algorithms except that ALGO-3 skips solving the optimization problem when there is no change detected in the network topology. If the per-slot traffic processing capacity of RRHs were 30, for example, both SDF and RND would cause the network congestion or the buffer overflow at some RRHs in any cases. On the other hand, it is not the case to the proposed schemes except when v=60 m/s.

Except one outlier occurring at v=60, the proposed schemes resulted in a smooth curve for all different values of *v*, even when *v* is large. It confirms that the proposed scheme can effectively respond to the degree of mobility of IoT devices. However, both SDF and RND show rather drastic curves especially when *v* is large, meaning that their performance is significantly dependent upon *v*. When v=60, there have been many time slots at which IoT devices are clustered. For this reason, all five algorithms resulted in having a relatively large value of the maximum load throughout the simulation. It is worth noting that the performance gap between SDF and RND becomes small when *v* is large. The high speed of IoT devices makes them spread out in the network quickly, therefore, the decision made by SDF becomes close to that of RND due to the uniform distribution of IoT devices in the given area.

**Figure 5:**
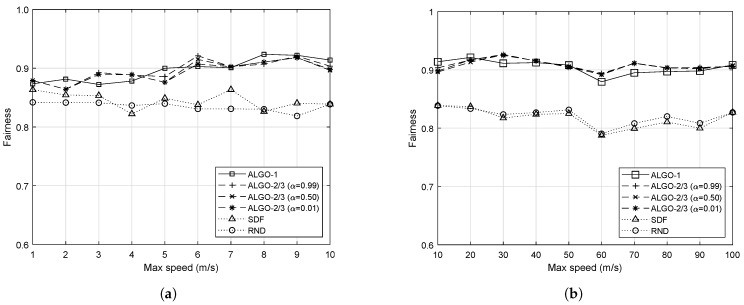
Average load balance performance measure by the Jain’s fairness index with respect to different upper bounds on the speed of 50 mobile IoT devices. (**a**) When IoT devices are moving at a slow speed; (**b**) When IoT devices are moving at a fast speed.

Next, we measured the precise load-balancing performance of those five algorithms. To do this, we used the Jain’s fairness index [31] which is one of the most widely used fairness index [32] for load-balancing analysis in wireless networks. Jain’s fairness index is defined as follows. For given *n* samples, $x_{1},x_{2},x_{3},\cdots ,x_{n}$, the fairness index is computed as follows:

(4) $$J\left(x_{1},x_{2},x_{3},\cdots ,x_{n}\right)=\frac{{\left(\sum_{i=1}^{n}x_{i}\right)}^{2}}{n\cdot \sum_{i=1}^{n}x_{i}^{2}},$$

where having 1 is the fairest and 1/n is the opposite. The fairness performance overview of the five algorithms with respect to different *v* is provided in Figure 5.

As expected from the previous results in Figure 4, the proposed schemes always outperform both SDF and RND, as shown in Figure 5. Although ALGO-1 outperforms ALGO-2/3 in some cases and vice versa, the difference is not significant. When *v* is small, i.e., from 1 to 3, performance of the five algorithms are quite close to each other. However, as *v* gets larger, the difference in the fairness performance becomes significant. The clustered mobility pattern shown at v=60 also resulted in a low fairness performance for all five algorithms. It is worth mentioning that both SDF and RND show very similar fairness performance especially when *v* is large, and it can be explained by the same reasoning as what we have provided: the uniformity of the IoT devices’ locations. It should be noted that different values of α do not cause any significant difference in the performance of ALGO-2/3.

**Figure 6:**
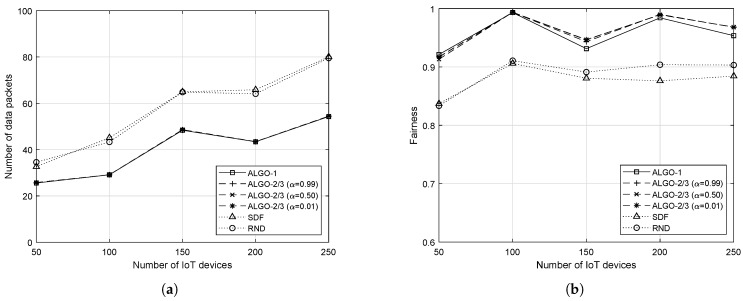
Average fairness performance with respect to different number of IoT devices when v=20. (**a**) Average maximum load; (**b**) Fairness performance.

We also conducted the same measurement with different number of IoT devices when v=20 m/s or, equivalently, 72 km/s. As it can be seen in Figure 6, the relative performance remains the same. ALGO-1/2/3 outperform both SDF and RND in terms of both the maximum number of packets to process by individual RRHs and the fairness index. Overall, the fairness performance of all five algorithms have been improved. Since the increased number of IoT devices randomly moves in the network, they tend to spread out evenly improving the fairness performance of both SDF and RND as well. However, the proposed schemes still achieve the highest fairness performance. In fact, for some cases, for example, such as when there are 100 and 200 devices in the network, the proposed schemes achieve close-to-perfect fairness. The increasing trend in Figure 6a is natural and expected, due to the increased number of IoT devices associated with each RRH on average.

### 4.3. Number of Handovers

**Figure 7:**
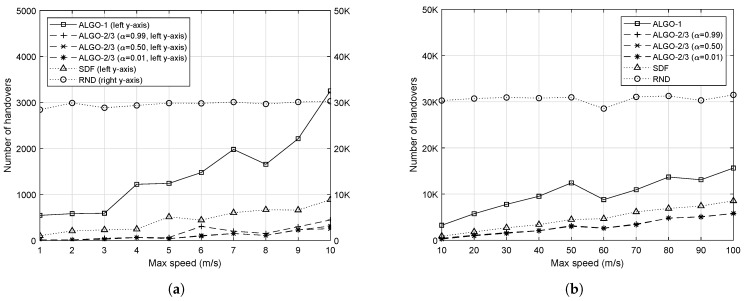
Total number of handovers with respect to different upper bounds on the speed of 50 mobile IoT devices. (**a**) When IoT devices are moving at a slow speed; (**b**) When IoT devices are moving at a fast speed.

We counted the total number of handovers that occurred with different values of *v*. Reducing the number of handovers is one of the main contributions of the proposed scheme, as it can prevent control message flooding. The results are depicted in Figure 7. In Figure 7a, RND corresponds to the right *y*-axis, while the left *y*-axis is for the other algorithms. As it can be clearly seen in Figure 7a, when *v* is small, RND incurs a significant number of handovers overall. In fact, that of RND does not change much throughout simulations with different values of *v*. This is because RND randomly chooses RRHs to associate with.

On the other hand, the other algorithms show gradual change as *v* increases. Obviously, ALGO-2/3 caused the fewest handovers due to the α-weighted penalty given to handovers. Moreover, ALGO-2/3 with the largest value of α resulted in having the most handovers among the same algorithms with different values of α. This is because its penalty to handovers is the least. It is worth mentioning that ALGO-1 resulted in more handovers than SDF. As SDF associates IoT devices with the closest RRHs, it suppresses handovers to some degree. On the other hand, ALGO-1 does not have such function reducing handovers. As long as the object function results in the optimal solution, it does not matter which IoT devices associate with which RRHs, as discussed in Section 3 (Figure 2).

**Figure 8:**
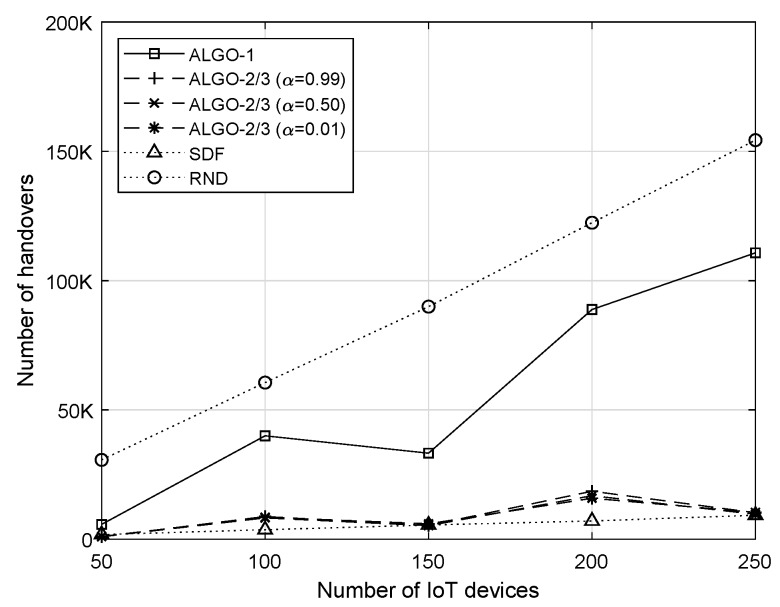
Total number of handovers with respect to the number of IoT devices when v=20.

When there are more IoT devices in the network, both RND and ALGO-1 show an increasing trend in the number of handovers, yet that of ALGO-1 is much less than that of RND, as shown in Figure 8. On the other hand, ALGO-2/3 and SDF keep the number of handovers small and stable. It is worth noting that SDF yields a smaller number of handovers than the other algorithms in most cases. When the network becomes overloaded, the effect of maximizing the fairness performance at the expense of handovers becomes noticeable. However, ALGO-2/3 result in the the number of handovers comparable that of to SDF in many cases with large number of IoT devices.

### 4.4. Time Complexity

**Figure 9:**
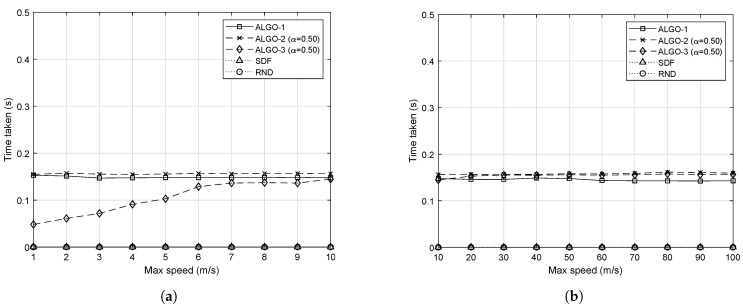
Average time taken to solve the given optimization problem with respect to different upper bounds on the speed of 50 mobile IoT devices. (**a**) IoT devices are moving at a slow speed; (**b**) IoT devices are moving at a fast speed.

The proposed algorithms allows finding optimal solutions for each time slot. Therefore, to enable the proposed methods running in real time, it is important to consider their time complexity. The average time taken in seconds to run each algorithm is shown in Figure 9. It should be noted that the plotted values, i.e., the time taken to solve the given optimization problem, include the time for making function calls for measuring time (In this work, we used tic and toc functions provided by MATLAB. We summed up the time required to run each algorithm for 1000 iterations, and then, we divided the sum by 1000 to get the average time.), modeling time for each given optimization problem, and time required for a solver to find the optimal solution (In CVX, for a given optimization problem, the modeling process runs first to transform the problem into the form that the particular solver can take in. After that, the solver solves the problem and then returns the optimal solution. [29]). Therefore, the actual time to solve the problem is smaller than the ones reported in Figure 9. In addition, if the operating platform is changed to a powerful computing machine, such as BBU pool in C-RAN, much less CPU time will be required to run the algorithms.

In short, solving SDF or RND takes a negligible amount of time compared to the proposed algorithms due to their simplicity. It takes almost the same amount of time to solve ALGO-1 and ALGO-2 because the corresponding optimization problems P. (2) and P.(3), respectively, are similar to each other except that P. (3) has some additional complexity for its relatively complex objective function. Although ALGO-2 and ALGO-3 are basically the same, and thus, take the same amount of time to find optimal solutions when IoT devices are moving fast, ALGO-2 is significantly efficient when the proximity matrix does not change often.

It is worth mentioning that the CPU time for SDF and RND took ≤1 ms in all cases. It is because the corresponding algorithms are quite simple. For each IoT device, the SDF algorithm compares the distance to nearby RRHs and chooses the closest one. On the other hand, for each IoT device, the RND algorithm randomly chooses an RRH among the nearby ones. However, the proposed schemes need to solve the optimization problem with binary variables. Owing to the advanced schemes available to reduce the time complexity of such problems, such as relaxation and branch-and-bound, solving binary (or integer in general) optimization problems does not take much time, as shown in Figure 9.

The CPU time values required for ALGO-1 and ALGO-2 are very close to each other because of the similarity in their problem formulation. However, ALGO-2 takes a longer time to compute because it has a more complex objective function. For a small *v*, ALGO-3 requires much shorter CPU time to run than ALGO-2 because the network does not change significantly when IoT devices are moving slowly, therefore ALGO-3 often skips the steps of solving the optimization problem. As *v* increases, the difference between the two becomes smaller. Overall, taking into account the limitations of the computing power of the PC used for this simulation and the difficulties in separating the actual solver time from the aggregated CPU time to find the optimal solution, the proposed algorithms can be used in real time if the same or similar network configuration is used, e.g., time slot of 1 s, tens of IoT devices, and a small data rate of IoT devices.

**Figure 10:**
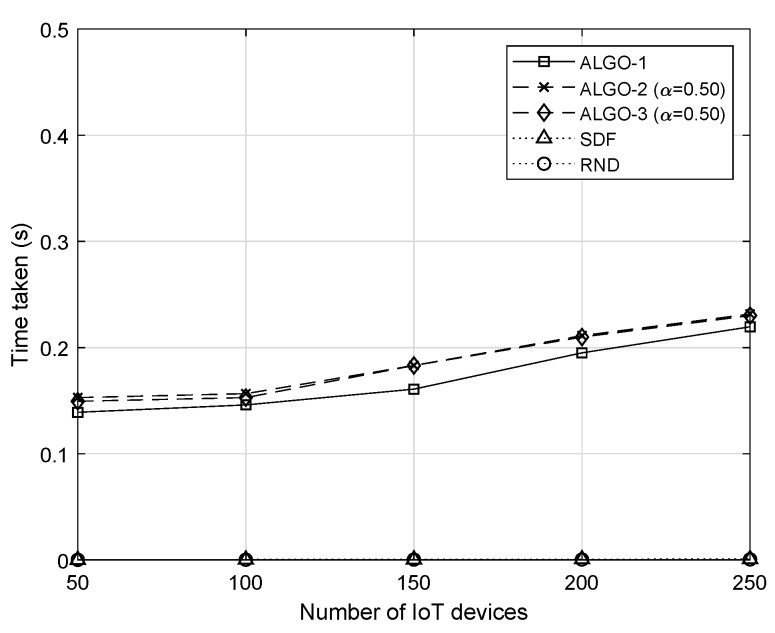
Average time taken to solve the given optimization problem when v=20.

Figure 10 shows the time complexity of the five algorithms in cases when there are more devices in the network. Even though the proposed methods need to solve computationally heavy optimization problems, the CPU time has not been drastically increased. Considering the configurations used for the evaluation, the time complexity has shown a rather linear pattern with a gentle slope. A further discussion on the time complexity is given in Section 5.

## 5. Discussion on Time Complexity

As shown in the previous section, the speed of IoT devices does not affect the time complexity significantly. The number of IoT devices in the network, on the other hand, increases the CPU time for solving the proposed optimization problems. This is because the increased number of IoT devices also increases the dimension of the decision variable X in the proposed problem formulation, hence increasing the problem complexity. We expect the same to happen if we increase the number of RRHs in the network. Although all different configurations tested in this study did not disturb the correct functioning of the proposed methods, it may not be the same in the case when there are much more IoT devices, e.g., thousands of IoT devices, working with much shorter time slots, e.g., ≪1 s.

One simple, yet effective solution we propose to overcome the possible limitation of the proposed schemes is *sectorization*. In a nutshell, the network divides the coverage area into multiple disjoint sectors, and then, runs the proposed methods for each sector separately in parallel. Although it is not always guaranteed to retrieve the location of IoT devices in real time, the location of RRHs are known in general. Therefore, we can easily identify which RRH belongs to which section. As each sector is separately handled from the rest, there should not be any IoT devices belonging to multiple sectors at the same time. Otherwise, an IoT device belonging to two sectors, for example, may end up having two associations at the same time, which is not allowed in this work.

**Figure 11:**
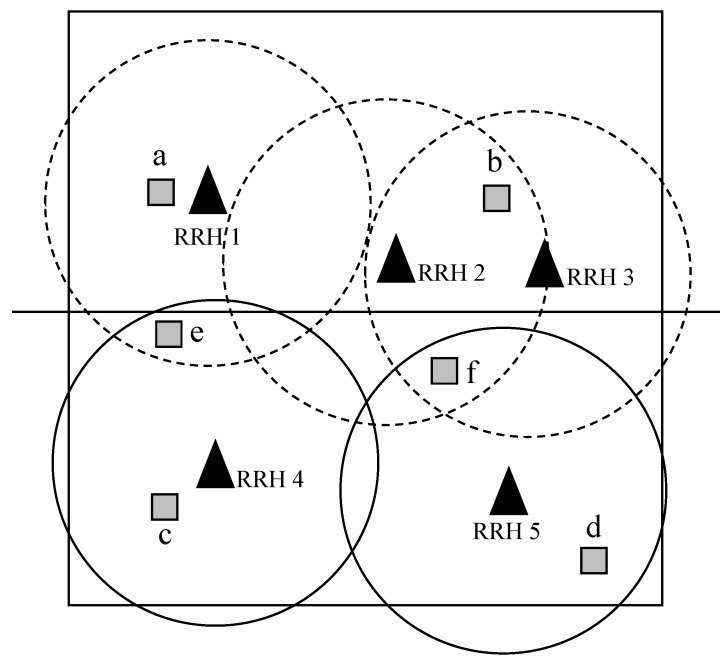
An example network with five RRHs from 1 to 5 and six IoT devices from *a* to *f*. The network is divided into two sectors, i.e., the upper and lower sectors, where RRHs 1–3 and 4–5 belong to the upper and lower sector, respectively.

To resolve this issue, we propose a probabilistic approach by using the number of RRHs nearby. As an example, let us consider the network as described in Figure 11. The area is 1000 m by 1000 m, and there are five RRHs marked as 1–5 and six IoT devices *a*–*f*. The coverage area of each RRH is shown by dashed lines. The location of each RRH is assumed to be known, while it is not the case for IoT devices. We divide the network into two sectors: upper half (denoted as *U*) and lower half (denoted as *L*). Given the location of RRHs, RRHs 1–3 are grouped into *U*, while RRHs 4 and 5 belong to *L*. IoT devices *a* and *b* belong to *U*, and *c* and *d* to *L*. However, it is not clear for *e* and *f*. Again, we do not assume their location to be known. For *e*, it is nearby RRH 1 in *U* and 4 in *L*. Using the proposed probabilistic algorithm, information on its proximity to *U* (i.e., to RRH 1) or *L* (i.e., to RRH 4) is replaced with probability 0.5 each. On the other hand, for *f*, information on its proximity to *U* (i.e., to RRH 2 and 3) or *L* (i.e., to RRH 5) is replaced with probability 1/3 or 2/3, respectively.

In this way, the dimension of the decision variable X is reduced from 5-by-6 to 3-by-4 for *U* and 2-by-2 for *L*. As the proposed algorithms optimize each sector separately in parallel, the time required to optimize the association for the entire network equals the CPU time to optimize the most complex sector. However, it is worth mentioning that the sectorization method does not yield the optimal solution, although there is a chance to achieve this. However, it is an effective way of reducing the time complexity of the proposed schemes, which might be essential for some applications where real-time operation is more important than yielding the optimal solution. We leave the further discussion on this as a future research topic.

## 6. Conclusions

In this work, we studied a C-RAN with large-scale IoT devices. We particularly focused on the optimal strategy of associating RRHs with large-scale, mobile IoT devices. To prevent the possible network congestion and control message flooding caused by unbalanced traffic load among RRHs and frequent handovers, respectively, we first proposed an optimal load-balancing user association strategy. By slightly revising the problem definition, thereafter, we propose an effective way of minimizing the number of handovers as well. We implemented a discrete time network simulator to conduct the performance evaluation, and comparison against conventional association algorithms. The results show that, in comparison to the conventional association schemes, the proposed methods allow balancing the traffic load effectively among RRHs overall and minimizing the number of handovers overall.

The proposed solutions in this work can be applied to general wireless network scenarios with densely deployed access points (APs). In addition to dense C-RANs, another possible example is the Wi-Fi or IEEE 802.11 network which is widely available nowadays. Due to their dense deployment, the proposed load balancing and handover minimization technique can significantly enhance the quality service for users. In addition, the proposed methods can also be used for networks that do not have dense APs as long as their transmission range is large, resulting in large coverage overlapping areas. Example networks are general cellular networks, Wi-Fi HaLow (or IEEE 802.11ah) [33], Sigfox, LoRaWan, and NB-IoT [34], to name a few.

As mentioned in Section 5, sectorizing or partitioning a given network into multiple disjoint areas to reduce the problem complexity is left as our future research direction. Using the decomposition theory to transform the proposed method into a distributed algorithm of a low complexity will also be considered in the future.

## Figures and Tables

**Table 1:** Summary of abbreviations.

Abbreviation	Meaning
BBU	BaseBand processing Unit
BS	Base Station
C-RAN	Cloud Radio Access Network
CAPEX	Capital Expenditure
IoT	Internet of Things
LPBS	Low-Power Base Station
LTE	Long Term Evolution
MBS	Macro Base Station
OPEX	Operating Expenses
QoS	Quality of Service
RRH	Remote Radio Head

**Table 2:** Summary of notations.

Notation	Meaning
N	Proximity matrix, $N={\left[n_{r,i}\right]}_{\left\{r\in R,i\in I\right\}}$
R	Index set of RRHs, {1,2,3,⋯,r,⋯}
I	Index set of IoT devices, {1,2,3,⋯,i,⋯}
$l_{i}$	Number of data packets to transmit by IoT device *i* to its associated RRH in a time slot
*c*	RRH’s data processing capacity (i.e., number of data packets per time slot)
$b_{r}$	Amount of background traffic (i.e., number of data packets) at RRH *r* at the given time slot

**Table 3:** Network parameters.

Parameter	Description	Value
|R|	Number of RRHs	15
|I|	Number of IoT devices	Between 50 and 250, inclusive
$l_{i}$	Traffic rate from IoT devices	Randomly chosen from {1,2,3} (pkts/slot)
*c*	RRH processing capacity	|I| (data packets per time slot)
$b_{r}$	RRH background traffic	Randomly chosen from $\left\{1,2,3,\cdots ,\frac{c}{2}\right\}$ (pkts/slot)
*v*	Velocity upperbound	Between 1 and 100, inclusive (m/s)
α	Weight used in ALGO-2/3 and P. (3)	0.99, 0.50, 0.01
